# A forensic-driven data model for automatic vehicles events analysis

**DOI:** 10.7717/peerj-cs.841

**Published:** 2022-01-05

**Authors:** Aymen Akremi

**Affiliations:** College of Computer and Information Systems, Umm Al-Qura University, Makkah, Saudi Arabia

**Keywords:** Vehicle detection, Forensics requirements, Semantic data model, Clustered Cameras network, Events analysis

## Abstract

Digital vision technologies emerged exponentially in all living areas to watch, play, control, or track events. Security checkpoints have benefited also from those technologies by integrating dedicated cameras in studied locations. The aim is to manage the vehicles accessing the inspection security point and fetching for any suspected ones. However, the gathered data volume continuously increases each day, making their analysis very hard and time-consuming. This paper uses semantic-based techniques to model the data flow between the cameras, checkpoints, and administrators. It uses ontologies to deal with the increased data size and its automatic analysis. It considers forensics requirements throughout the creation of the ontology modules to ensure the records’ admissibility for any possible investigation purposes. Ontology-based data modeling will help in the automatic events search and correlation to track suspicious vehicles efficiently.

## Introduction

Recent years have witnessed an increasing number of violent events occurring all around the world (Organization, 2014). Security services are in continuous work and at utmost readiness for anticipating eventual crimes by identifying suspicious vehicles used for illegal purposes. In most cases, the vehicles are used by criminals to spy, hit, transport, and escape from justice. Therefore, security forces improve the security provisions in checkpoints and sensitive roads using cameras. The efficiency enhancement of cameras monitoring the inspection points in identifying suspicious targets goes through the suitable models and approaches used for image recognition and classification. It is of paramount importance to connect all checkpoints and share relevant information through enhanced communication techniques to improve recognition performance. Thus, the database of each checkpoint will benefit from the cumulative extracted data and analysis of others. For instance, the collected critical details of an escaped vehicle from an inspection point will be immediately communicated with others, enabling fast processing of the detected images and, therefore, taking the required policies against that vehicle before escaping the next checkpoint.

Rapid data processing and sharing present the main factor for effective checkpoint traffic control and management while preserving the collected data’s admissibility. Its importance increases significantly in crowded cities such as Makkah (https://www.britannica.com/place/Mecca), where checkpoints are the most valuable methods to filter the vehicle of the worldwide coming pilgrims for Hajj (https://www.britannica.com/topic/hajj) and Umrah (https://www.britannica.com/topic/umrah). Thus, adopting a suitable forensic-aware data modeling schema will enhance the storage, analysis, and sharing process, ensuring that the court will not refute the gathered data from any checkpoint. Images, for instance, are relevant evidence whenever it keeps their admissibility. Thus, the data model must consider further forensics and investigation purposes.

To sum up, the different issues this paper aims to resolve are related to the significant data communication, real-time data analysis architecture, and the forensically sound and automatic data analysis to extract relevant information (more details in Section 2).

This paper deals with these issues by presenting a scalable and efficient framework for data communication and management between different parties in the vehicle recognition checkpoint. Also, it proposes a new ontology-based data model to automatically and forensically sound deduce relevant information. Mainly, it is a two-fold approach consisting of:

1. A communication control and management design architecture. This effort proposes a Cluster-based architecture where each checkpoint presents a self-contained cluster that processes their local data and shares only relevant information with other locations. The proposed model uses a central Cloud-based database to collect and keep trails about the information gathered from all checkpoints.
2. A forensically sound data modeling between the various system units. The paper also presents a new ontology-based forensically sound data model for managing and analyzing the massive collected data. It includes all related specifications and concepts to vehicle recognition, frauds, and incidents within checkpoints using surveillance cameras. Then, it uses the ontology-based model to track suspected vehicles, using Semantic Web Rule Language^1^ 1SWRL: A Semantic Web Rule Language Combining OWL and RuleML: http://www.w3.org/Submission/2004/SUBM-SWRL-20040521
, as a test scenario.

The remainder of this paper is divided as follows. Section 2 depicts checkpoints’ constraints and requirements. In Section 3, the paper presents and details the multi-camera management framework and depict the limitation of other design choices. Section 4 shows the proposed forensically sound ontology-based data model for vehicle image recognition and analysis. Then, a test demonstration scenario using SWRL rules to track and analyze suspicious cars is presented. In Section 5, the paper discusses several existing related works dealing with multi-cameras management and data modeling, especially those considering forensics requirements. Finally, Section 6 concludes the article.

## Case study definition and challenges of multi-Cameras managements

This section defines the case study for which the proposed forensics-aware data model is designed. The study aims to address the issues related to multiple cameras management in several security checkpoints. More specifically, it seeks to tackle the problems associated with controlling and managing the data gathered from various distributed inspection points in a forensically sound manner. This research is motivated by the current and increased need for Makkah’s security services to effectively handle the big data received from the various inspection points. Thus, this paper presents the checkpoint system specification and the different challenges related to the required devices, processing speed, and the data size before creating the model.

The checkpoint system consists of several static cameras installed in several checkpoints containing multiple roads. Each vehicle is scanned by two digital cameras and one 3D camera. The used checkpoint’s cameras are of type grasshopper 2.0 MP color firewire (camps://www.flir.com/support/products/firewire-cameras). They provide a 1624 ×1224 resolution image with a 30 frame rate. The camera has a 14-bit analog to digital converter (ADC) and 32 MB image buffer. The power consumption is 3.5 W at 12V. Each car requires 3 s to be fully inspected, producing an image size of about 2 MB. The images should be carefully stored for possible security and forensics needs after analysis and possible detection of suspicious vehicles. Since critical information may be identified from the data received, checkpoints require reliable communication to share information and improve detection performance. It is mandatory to consider during the cameras selection the different source cameras models to enhance the forensics requirements preservation (Amerini et al., 2021).

The above case study presents several challenges related to camera management, fast real-time vehicle recognition, information sharing between checkpoints, and rapid analysis of events from multiple distant locations while considering forensics constraints. The main challenges are depicted as follows:

• **Data processing scalability.** Concerned with the checkpoint’s control system’s ability to respond to any considerable number of vehicles accessing them in terms of data processing and storage scalability. For instance, during the Hajj season in Makkah, the roads witness an exponential increase in vehicle number. The checkpoints must process them reliably and detect any violation.
• **Information and network security.** It increases depending on the data sensitivity transferred between involved parties. Information such as car plate number, color, type, checkpoint passing time, and maybe the driver’s general description must not be altered or modified during their transmission.
• **Information sharing.** It is about the fast, secure, and forensically sound exchange of information between different checkpoints, which will reduce the processing time and improve the security service agent’s readiness.
• **Forensically sound data processing.** It aims to preserve the gathered data’s admissibility for any further investigation need. The system must adhere to forensics rules without decreasing its performance.

To deal with the above challenges, this study proposes a two-fold approach that consists of Cluster-based checkpoint design and forensically sound data modeling, detailed in the following sections.

## Cluster based checkpoint’s multi-Cameras management framework

**Figure 1:**
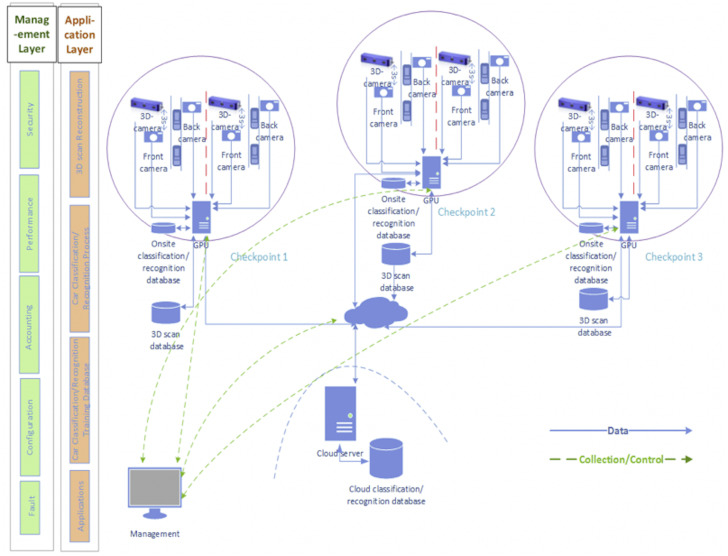
Checkpoint system management architecture.

The design of the Cluster-based framework is driven by the required specifications mentioned in section II. Figure 1 presents the different architectural ingredients. The framework takes advantage of the distributed architecture as follows:

• the replication and independence of databases, processing units, and network devices increases transaction reliability, availability, and fault tolerance.
• the enhanced modularity of distributed architectures enables the easy modification of the distributed database without affecting other system’s modules or causing scalability issues.
• the analysis load distribution into several processing units enables an improved performance to handle and process big data.

### Framework ingredients

The proposed checkpoint’s vehicle design encompasses two layers:

#### Management layer

The management layer deals with system management issues. It adopts the ISO telecommunications management model FCAPS (Fault, Configuration, Accounting, Performance, and Security) to organize the network management functions into five categories covering all telecommunications issues (Goyal, Mikkilineni & Ganti, 2009). The FCAPS model is widely used in big organizations to manage any networked system (Kwiecień & Opielka, 2012). This paper adopts best practices in the literature (Widjajarto, Lubis & Syahputra, 2019) to implement and realize the framework.

#### Application layer

The application layer includes application specifications and requirements. According to the case study specification, the application requirements are divided into four phases. Each phase has specific tasks distributed in the architecture according to each task requirement. The phases are:

• 3D scans reconstruction phase: this phase reconstructs the 3D scans based on the images and shapes provided from the camera and 3D scan devices, respectively. The scanning process takes 3 s and delivers 2 Mb for each vehicle. After capturing the 2D images scans, the data are transmitted to the onsite Front-End layer processing units (GPU-based server) to reconstruct the 3D shapes in real-time using monocular methods (Zollhöfer et al., 2018; Russell, Yu & Agapito, 2014). The system must adhere to the best practices in handling data to maintain their admissibility once required in a possible investigation case. Section 4 presents the proposed data model to ensure the integrity of the data chain of custody.
• Car Classification/recognition phase: the application classifies each car using accurate classification algorithms executed by the GPU to determine the car class based on the different classification features.
• Car recognition/classification training database: the car recognition database includes images of car models or any photograph of a suspicious car. It has a non-deterministic size and should be shared between all checkpoints. Thus, this study adopts a central Cloud recognition database shared between all checkpoints (clusters) to deal with scalability requirements. A Cloud server manages the training database for any updating or synchronization requirements.
• Application phase: deals with the management of the different architectural components.

#### Processing architecture

**Figure 2:**
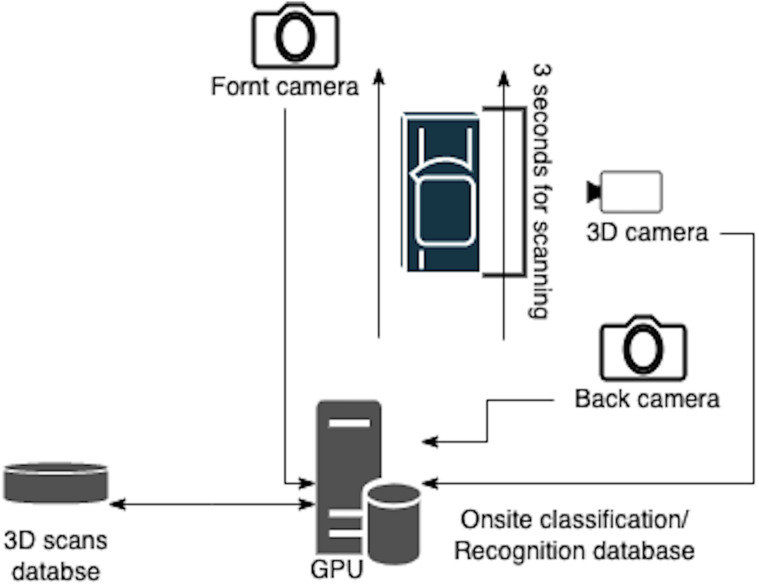
Front-end cluster.

The proposed framework encompasses two processing layers based on the detection and analysis time, which are:

• Front-End layer is represented by each Cluster (checkpoint) detailed in Fig. 2. It deals with real-time 3D scan reconstruction, car classification, recognition, and onsite data storage. The used classification algorithms must also consider real-time requirements to enable fast suspicious car detection. Mainly the used algorithms are finite gamma mixture based method (Al-Osaimi & Bouguila, 2015), beta-Liouville and Dirichlet distribution based methods (Fan et al., 2016), and Pitman-Yor process mixtures of Beta-Liouville Distributions method (Fan, Al-Osaimi & Bouguila, 2016).
• Back-End layer is a cloud-based solution responsible for storing and maintaining the recognition/classification training database. The back-end layer includes the management unit, which controls all connected clusters and receives their status to intervene in any failures.

## Forensically sound semantic data modeling Schema

To enhance the data integration, offer a reliable, extensible content description, and fit the need for automated vehicle tracking and analysis, the paper adopts Semantic Web technologies; namely, it uses ontology. This technology improves data sharing between the different checkpoints and integrates heterogeneous resources of various hardware and software technologies. The ontology also allows the deduction of new details such as finding contradictions or validating things through the use of reasoning engines such as Pellet (https://www.w3.org/2001/sw/wiki/Pellet). This feature is used to automatically track and identify the suspicious vehicles deduced by the reasoning engine.

The key feature of the proposed data model resides in its easy integration with the existing tracking system through the use and extension of current standards. Using standards is required to increase its interoperability and integration within already implemented vehicle tracking systems. Thus, this paper creates a semantic data model by reusing several existing ontology standards and selecting only the suitable ontology modules related to the research topic. Then, it adds and completes the different required classes and relations specific to the case study based on scoping and tailoring techniques. During the scoping process, this effort distinguishes three relevant standards and researches to be adopted. Mainly they are:

• The Incident Object Description Exchange Format (IODEF) (Danyliw & Takeshi, 2016).
• The Road Accident Ontology (Dardailler, 2012).
• The Vehicular Accident Ontology designed to Improve Safety on the Roads(VEACON) (Barrachina et al., 2012).

Then, the paper proceeds for the tailoring process to select only associated-relevant items and propose missing elements and modules as required. Thus, every established ontology module is either newly proposed or extracted from existing efforts and extended by new attributes. This process aims to ensure the integration of forensics requirements into the ontology without negatively impacting the performance and reliability of the vehicle tracking system.

Table 1, depicts the different selected standards and research efforts and their adaptation tailoring process. Also, it shows the newly proposed modules and extensions to existing standards.

**Table 1:** The scoping and tailoring process used for the Standards selection and tuning.

Ontology modules	Scoping (selected standard)	Tailoring (Proposed, Mapped, Filtered)	Description
Checkpoint		Proposed	provides information about checkpoints
Technologies		Proposed	describes all used technologies
Assessment		Proposed	used types to asses the incident impact. The different impact types are covered by the impact module
Impact		Proposed	covers the diffrent type of incdient impact
Vehicle	VEACON ontology (paper)	Filtered + Extended	describes the vehicles
Fraud	Road Accident Ontology (W3C standard)+ VEACON ontology (paper)	Filtered + Extended	provides information about vehicle accidents attributes and frauds types
Contact	RFC Standard (part of the IODEF and RID mapped to OWL)	Filtered + Mapped	describes the different concepts and attributes of contacts
Record	RFC Standard (part of the IODEF and RID mapped to OWL)	Filtered + Mapped	provides all information about the records captured from any checkpoint or delivered by any resource
Incident	RFC Standard (part of the IODEF mapped to OWL)	Filtered + Mapped	provides all information about the detected incidents and the events associated with each incident
Security	RFC Standard (part of the IODEF and RID mapped to OWL)	Extended	Provide information of the security attributes related to the transfer and alteration of the data

**Figure 3:**
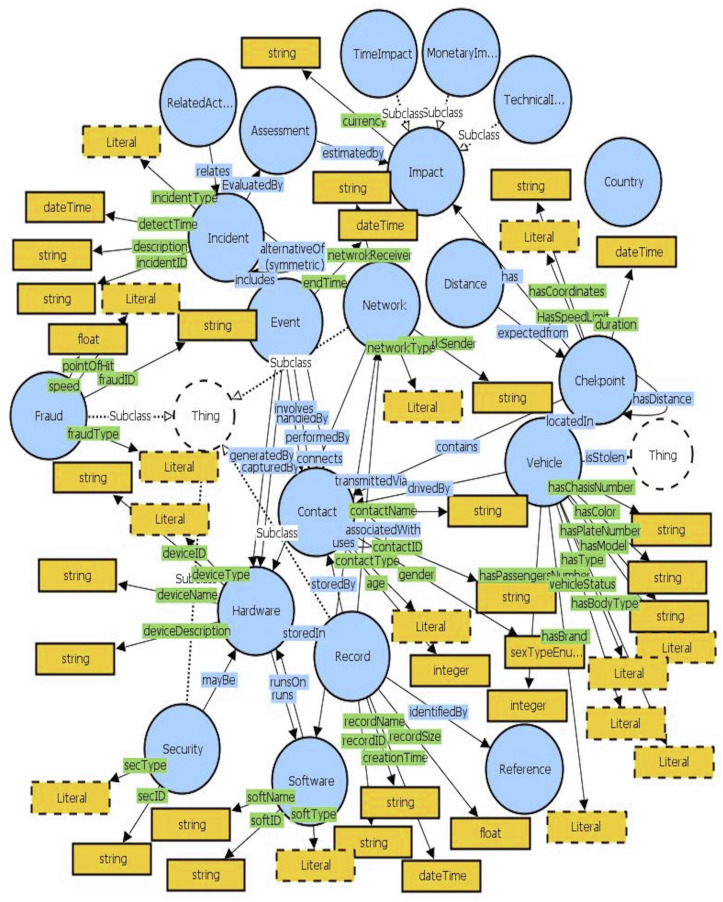
Forensics-aware checkpoint’s vehicle recognition ontology.

The proposed ontology encompasses several components covering all activities and used technologies related to vehicle control inspection points. Also, it considers forensics requirements throughout the design of the semantic-based data model. Figure 3 presents the overall proposed ontology named Forensics-aware Checkpoint’s Vehicle Recognition Ontology (FCVRO).

### FCVRO modules overview

The following includes the details of the main ontology modules showing their goals and advantages. Several new ontology modules depicted in Table 1 are not described since their purpose could be easily estimated from its naming.

#### Vehicle modeling

The vehicle module (see Table 2) encompasses all attributes that distinguish a vehicle from another. It determines whether the car is self-driving (auto) or not (nauto). Also, it determines the legal status of the vehicle. This module communicates with the Contact module since each vehicle has a driver and eventual passengers and checkpoint module where the vehicle is located and is subject to the recognition process.

This paper treats only cars vehicles since, currently, it implements only algorithms dealing with car processing.

#### Contact modeling

The purpose of the Contact module is to model all human beings in contact with the recognition process. Contact may be the police agents, system administrators, vehicle drivers, and any possible person that may impact the final recognition process. The contact module (see Table 3) is connected directly with the vehicle (in this case, the contact may be the driver or passengers), the fraud (the person(s) that commits the scam), and the event (the person(s) involved in the events) classes. Also, the contact module includes all persons working in the checkpoints and those maintaining the recognition system.

#### Incident & event modeling

This module includes Incident and Event classes (see Table 4). Each incident may have one or several events. An event is the smallest complete task that occurs by an active part. The incident describes all actions and events within checkpoints and/or within the intermediate systems and tools. Thus, an incident may cover several events generated from different checkpoints or/and medians. An incident may be internal (caused by internal contact) or external (caused by external contact such as a new suspected vehicle).

**Table 2:** Vehicle module.

Object class	Data properties	Description
Vehicle	hasType	whether it is self-driving vehicle(auto) or not (nauto)
Vehicle	hasChasisNumber	determines the number of the vehicle chassis
Vehicle	hasPassengerNumber	determines the number of passengers in the vehicle in the recognition moment
Vehicle	hasBrand	determines the brand of the vehicle
Vehicle	hasBodyType	which are sedan, pick-up or fan
Vehicle	hasModel	the year of the vehicle manufacture
Vehicle	hasColor	the color of the vehicle
Vehicle	hasPlatePlate	the plate number of the vehicle
Vehicle	vehicleStatus	wether the vehicle is wanted or nothing
Vehicle	SpecUse	vehicle special use category applied

**Table 3:** Contact module.

Object class	Data properties	Description
Contact	contactID	presents the unique identifier of the contact
Contact	contactName	name of the contact
Contact	contactType	all possible contact category involved in recognition event such as police agent, driver...
Contact	gender	gender of the contact
Contact	age	age of the contact

**Table 4:** Incident module.

Object class	Data properties	Description
Incident	incidentID	incident unique identifier
Incident	startTime	incident start time which the detect time of the first event
Incident	endTime	incident end time which is the end time of the last event
Incident type	incidentType	wether the incident causer is internal , external or both
Incident	description	incident global description
Event	eventID	event unique identifier
Event	detectTime	event detect time
Event	endTime	event end time
Event	eventType	wether the event causer is internal or external
Event	description	event description

Each incident is evaluated *via* the assessment class to determine its monetary, time, technical loss, and severity impact. Since forensics requirements differ from one county to another, the assessment class could be adapted and extended by new forensics metrics using metrics elicitation frameworks that reflect the country’s regulations (Akremi, 2021a). The incident module has connections with the Contact, Fraud, Record, and Technologies modules.

#### Technologies modeling

The technologies module (see Table 5) covers all classes and attributes related to the system’s hardware and software tools. Mainly, it distinguishes three categories: Hardware (encompasses the different hardware elements such as cameras, routers, computers, etc.), software (includes all software requirements such as recognition and transmission methods, soft security tools), and network (defines the various used network interfaces and the different connections as well as the used technologies).

**Table 5:** Technologies module.

Object class	Data properties	Description
Software	softID	software unique identifier
Software	softName	software name
Software	softType	software category type
Hardware	deviceID	unique hardware identifier
Hardware	deviceName	hardware name
Hardware	deviceDescription	hardware description
Hardware	deviceType	hardware category
Network	networkSender	network sender side
Network	networkReciver	network receiver side
Network	networkType	network type as wire or wireless

The technology module components are secured using the security module provisions. It is essential to use trusted software to integrity-aware process the gathered data to avoid admissibility issues. A good solution is their validation using code review tools (Akremi, 2021b) before deployment.

#### Records and security modeling

The record module (see Table 6) mainly deals with the forensically sound processing and preservation of generated files and data for further use. Within the record module, the forensics requirements in the ontology design are incorporated and ensured through security techniques. The aim is to guarantee the admissibility of records for possible use by the court.

**Table 6:** Record and security modules.

Object class	Data properties	Description
Record	recordId	record unique identifier
Record	recordName	record name
Record	recordSubject	The topic and brief record description
Record	recordSize	record size
Record	creationTime	record creation time
Security	secID	unique identifier of security provision
Security	secType	type of security provisions for example detective, corrective, detterent, preventive,etc
Security	description	security provision type description

Based on digital forensics researches (Casey, 2018) and standards; a record is admissible when it preserves its ***authenticity*** through the preservation of records identity and integrity (Akremi et al., 2020; Duranti & Rogers, 2012), ***privacy*** by avoiding any kind of private information breaches during the data seizure (Akremi et al., 2020), ***comprehensiveness*** by ensuring that no missing information exists in the final report (Grobler, Louwrens & von Solms, 2010), ***relevance*** by focusing on presenting only evidence pertinent to the case), and ***not being hearsay*** since *”electronic documents generated and made in the usual and ordinary course of business are not hearsay”, Duranti2012trust*. The proposed ontology models the admissibility requirements of the record *via* both the Record and Security modules.

The record module is connected to the contact handling or generating the record, the hardware that may create, handle, or store the documents, the security provisions to grantee the admissibility and security of records, and the communication network responsible for their safe and reliable transmission.

The Security module’s purpose is to provide all required security provisions for other modules (see Table 6), including software or hardware tools. Aside from protecting the system from malicious and hacker penetration, this module provides the means to forensically sound record processing and preservation, such as delivering integrity techniques (MD5, Sha1, etc.).

#### Fraud modeling

The fraud module (see Table 7) describes the various possible frauds the system aims to identify. It is connected only with the event class to determine the event fraud type.

**Table 7:** Fraud module.

Object class	Data properties	Description
Fraud	fraudID	Unique identifier of the committed fraud
Fraud	fraudType	fraud type which may be a theft, hit, licence plate, or etc
Fraud	description	fraud type description
Fraud	pointOfHit	determines the point of impact
Speed	speed	determines the speed of the vehicle when it exceeds the speed limit.

### Automatic fraudulent vehicles detection using the ontology

The proposed ontology’s main objective is to automatically and forensically sound validate or omit a possible hypothesis about vehicle suspicion based on real-time verification of already stored information and current captured data. The idea is to infer SWRL rules that define facts and possible recognition patterns over the ontology and verify the proposed hypothesis’s conformity. This paper describes three scenarios of vehicle frauds and automatically identifies them when the frauded vehicle passes through checkpoints and immediately alerts the authorities reliably and securely. These scenarios are:

1. Vehicles that may be stolen.
2. Vehicles that may have a fraudulent license plate.
3. Vehicles that may be involved in a hit and run accident.

The paper presents the definition and implementation of the different SWRL rules enabling the validation of each scenario’s hypothesis through their inference over the proposed ontology. Then, it uses the PELLET reasoning engine (Sirin et al., 2007) to extract and fire rules over the proposed ontology. Therefore, the reasoner will determine the defined rules’ satisfaction and notifies the administrator by any deduced event. Table 8 describes the variables used by the OWL rules.

**Table 8:** SWRL variables description.

Variable name	Description
v	refers to a Vehicle of type Object
a	Refer to the type (autonomous or not-autonomous) of v
c	Refers to the color of v
ct	Refers to the individual driving v
m	Refers to the model of v
t	Refers to the body type of v
p	Refers to the license plate number of v
g	Refers to the gender of ct
ch	Refers to a checkpoint of type Object
d	Refers to the passing time of v through ch
i,j	Refers to incidents of type Incident
e1,e2	Refers to events of type Event
ac	Refers to an activity
f	Refers to a fraud of type object

The object properties used within OWL rules are:

• isStolen–holds when a vehicle is identified/deduced as stolen.
• hasFraudLicensePlate–holds when a vehicle is identified/deduced as having an illegal license plate.
• isHitRun–holds when a vehicle is identified as running away after a hitting accident.
• loactedIn–holds when the vehicle is identified at a checkpoint.
• relatedTo–holds when two incidents have relation to the same activity.
• happenedBefore–holds when an event happens before another event.

#### Scenario 1 - vehicles that may be stolen

**Figure 4:**
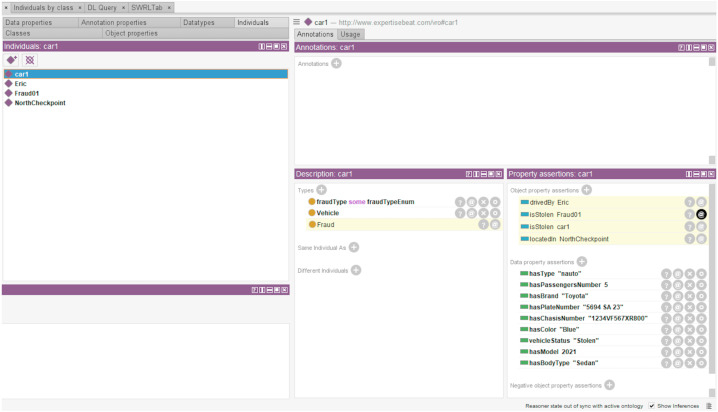
Vehicles that may be stolen rule execution and resulted inferred knowledge.


 
Identifying stolen vehicles is a daily police mission since this type of fraud is widely committed. Based on some already collected or calculated data, the objective is to identify stolen cars passing the checkpoints. For instance, the SWRL rule in Listing 1 identifies any none self-driving vehicle with a blue color, 2021 model, sedan body type, and a ”5694 SA 23” license plate number. These data are provided to the system *via* a graphical interface to enable vehicle searching and editing. Figure 4 shows the inferred knowledge after executing the SWRL rule 1. It shows the generation of new information about the car owner’s name, the checkpoint where the car was detected last time, and the fraud number associated with the car theft.

      Listing 1: SWRL rule of stolen vehicles 
Vehicle (?v)ˆhasType (?v ,  ?a)ˆ hasColor (?v ,  ?c )ˆ Contact (? ct )ˆ drivedBy (?v ,  ? ct )ˆ 
Fraud (? f )ˆ fraudType (? f ,  ” Stolen ”)ˆ gender (? ct ,  ”Male”)ˆ hasModel (?v ,  ? m )ˆ 
hasBodyType(?v ,  ? t )ˆ hasPlateNumber (?v ,  ?p)ˆ swrlb : equal (?a ,  ”nauto ”)ˆ 
swrlb : equal (?c ,  ”Blue ”)ˆ swrlb : equal (?m,   2021)ˆ swrlb : equal (? t ,  ”Sedan ”)ˆ 
swrlb : equal (?p ,   ”5694  SA  23”)  −>  isStolen (?v ,  ? f )    


#### Scenario 2 - vehicles that may have a fraud license plate

Same as the stolen vehicle rule, identifying cars with fraudulent license plates are based on the same data. The system will then compare them with any data extracted from passing cars and notify the administrator about wanted ones. The rule in Listing 2 determines a none self-driving car with a fraudulent license plate number.


 
                      Listing 2: SWRL rule of fraud vehicle license plate 
Vehicle (?v)ˆhasType (?v ,? a)ˆ hasColor (?v ,? c )ˆ Contact (? ct )ˆ drivedBy (?v ,? ct ) 
ˆgender (? ct ,” Male”ˆˆxsd : string )ˆ hasModel (?v ,?m )ˆhasBodyType(?v ,? t ) 
ˆFraud (? f )  ˆfraudType (? f ,  ” Stolen ”)ˆ swrlb : equal (?a ,” nauto”xsd :ˆˆ string ) 
ˆswrlb : equal (?c ,” Grey”ˆˆxsd : string )ˆ swrlb : equal (?m ,”1999”ˆˆ xsd : decimal ) 
ˆswrlb : equal (? t ,” fan ”ˆˆxsd : string)−>  hasFraudLicensePlate (?v ,? f )    


#### Scenario 3 - vehicles that may be involved in a hit and run accident

In this scenario (see Listing 3), cars involved in hit accident and run from the accident scene is modeled. In this case, the vehicle is searched based on some information provided by witnesses and already installed control cameras. The search is focused on the checkpoints that the driver may pass through. Each time, the system compares the detected time of cars passing the checkpoint with the accident reporting time received from other checkpoints. It processes only vehicles that arrived in or after the determined accident time. Finally, the system notifies the police agents about the detection of any suspected car.

The rule in Listing 3 uses the following variables; Local: determines the accident coordinates, Color: the color of the vehicle, BodyType: vehicle body type, Model: vehicle manufacturing year, LicensePlate: full or partial vehicle license plate, Gender: the driver gender, and HitTime: the accident time.

 
                            Listing 3: SWRL rule of hit and run fraud 
Vehicle (?v)ˆ hasColor (?v ,? c )ˆ Contact (? ct )ˆ drivedBy (?v ,? ct )ˆFraud (? f ) 
ˆfraudType (? f ,  ” Stolen ”)ˆ gender (? ct , g)ˆ hasModel (?v ,?m )ˆhasBodyType(?v ,? t ) 
ˆhasPlateNumber (?v ,? p)ˆ swrlb : equal (?c , Color )ˆ swrlb : equal (?m, Model) 
ˆswrlb : equal (?p , LicencePlate )ˆ swrlb : equal (? t , BodyType)ˆ swrlb : equal (?g , Gender) 
ˆCheckpoint (? ch )ˆ locatedIn (?v ,? ch )ˆ Location (? Local )ˆ DurationexpectedFrom (? Local ,? ch ) 
ˆ duration (?ch ,? d)ˆ swrlb : greaterThanOrEqual (?d ,? HitTime)−>  isHitRun (?v ,? f )    

### Events tracking and research

The events research and tracking module aim to help traffic control system users such as investigators and security agents identify information quickly and deduce new relations between data through tasks automation. Also, inferring over semantic data representation will help to detect the low-level tracking algorithm errors, which enhances the overall system performance (Greco et al., 2016).

#### Events tracking and ordering

The following rules aim to link the events from the same hardware resources in some checkpoints and order the aggregated events associrelatwithto the same oreparate incidents.

The rule in Listing 4 verifies the possible relation between two incidents to the same activity(RelatedAcivity). The activity is identified by a car driver (an actor) crossing the checkpoint with a specific address. Comparing different detected addresses of different drivers may reveal the affiliation of the incidents to the same related activity and being considered alternatives.

 
                            Listing 4: SWRL rule of hit and run fraud 
Incident (? i )ˆ relatedTo (? i ,? actv1 )ˆ attributes (? actv1 ,? driver1 )ˆ resides (? driver1 ,  ?n) 
ˆhasAddress (?n ,  ?d)ˆ Incident (? j )ˆ relatedTo (? j ,  ? actv2 )ˆ   attributes (? actv2 ,  ? driver2 ) 
ˆ resides (? driver2 ,  ?n1)ˆ hasAddress (?n1 ,  ?d1)ˆ vlan− num(?d ,  ?c )ˆ vlan− num(?d1 ,  ?c1 ) 
ˆswrlb : equal (? c1 ,  ?c )ˆ DifferentFrom (? i ,  ? j)−>alternativeOf (? i ,  ? j )    

The rules in Listings 5 and 6 use the OWL new defined object property ”happened before” to order the events associated with the same or multiple incidents. The ”happened before” object property uses the event detection time to determine events order.

 
              Listing 5: SWRL rule of events ordering of the same incident 
Incident (? i )ˆcompromisedBy (? i ,? e1 )ˆcompromisedBy (? i ,? e2 )ˆ Event (? e1 ) 
ˆdetectTime (? e1 ,  ? t1 )ˆ Event (? e2 )ˆ detectTime (? e2 ,  ? t2 )ˆ DifferentFrom  (? e1 ,  ?e2 ) 
ˆswrlb : lessThanOrEqual (? t1 ,  ? t2)−>happenedBefore (? e1 ,  ?e2 )    


 
     Listing 6: SWRL rule of events ordering belonging to different incidents 
Incident (? i )ˆ Incident (? j )ˆcompromisedBy (? i ,  ?e1 )ˆcompromisedBy (? j ,  ?e2 ) 
ˆEvent (? e1 )ˆ detectTime (? e1 ,  ? t1 )ˆ Event (? e2 )ˆ detectTime (? e2 ,  ? t2 ) 
ˆDifferentFrom  (? i ,  ? j )ˆ DifferentFrom  (? e1 ,  ?e2 )ˆ lessThanOrEqual (? t1 ,  ? t2 ) 
−>happenedBefore (? e1 ,  ?e2 ) 


Inferring SWRL rules using reasoning inference tools (*i.e.,* Pellet) creates new links between events and automatically deduces new knowledge in near real-time.

#### Events research

Since the size of the collected data by checkpoints is significant and in continuous increase, this paper defines SPARQL (Steve Harris, Seaborne & Consortium, 2013) (Protocol and RDF Query Language) queries for extracting partial researched data from the ontology as a tree to limit the research area. The queries syntax of SPARQL uses similar clauses as SQL with the advantage of enabling the querying of semi-structured data from multiple heterogeneous local or remote sources. Consequently, it improves the research time aside from automating the research task by predefined queries for specific objects. The first query in Listing 7 constructs a graph containing the queried data (*i.e.,* Eric’s car driver in this query instance).


 
         Listing 7: SPARQL query that extracts partial queried data graph 
PREFIX  rdfs :  <http ://www.w3. org /2000/01/ rdf− schema #> 
PREFIX  rdf :<http ://www.w3. org /1999/02/22− rdf− syntax− ns #> 
PREFIX  vro :  <http :// expertisebeat . com/ VRO #> 
  construct {?e1  vro : involves   vro : Eric . 
    ?e1  vro : flows  ?s1 . 
    ?s1  vro : runs  ?v1 . 
    ?e1  vro : contains  ?e2 . 
    ?e2  vro : contains  ?e3 .  ?e3  vro : flows  ?s3   .  ?s3  vro : runs  ?v3 .} 
 where{?e1  vro : involves   vro : Eric   . 
    ?e1  vro : flows  ?s1 . 
    ?s1  vro : runs  ?v1 . 
    ?e1  vro : contains  ?e2 . 
    ?e2  vro : contains  ?e3 .  ?e3  vro : flows  ?s3   .  ?s3  vro : runs  ?v3 .}     The second query in Listing 8 is executed over the graph generated by the first query. It selects subjects, predicates between them, and objects (instances of Eric’s car driver in this query) that exist in the graph.

 
    Listing 8: SPARQL query executed over the first graph in Query 1 
PREFIX  rdfs :  <http ://www.w3. org /2000/01/ rdf− schema #> 
PREFIX  rdf :<http ://www.w3. org /1999/02/22− rdf− syntax− ns #> 
PREFIX  vro :  <http :// expertisebeat . com/ VRO #> 
  s e l e c t   ? s  ?p  ?o . 
  where{? s  ?p  ?o .}    

## Related works and discussion

This paper reviewed the existing efforts dealing with video surveillance data representation for self and non-self-driving vehicle tracking and their solutions to address scalability, big data search, and forensically sound records processing. According to the literature review, any ontology-based representation does not exist encompassing all proposed forensics-aware checkpoint’s vehicle recognition ontology modules. However, only a few research sets are identified tackling video event descriptions, events tracking, and scalability issues.

Mostfa & Ridha (2019) proposed a vehicle plate number recognition through a distributed camera-based subsystem installed in several checkpoints connected to a central database. They could detect license plates effectively within 2 to 4 s. However, their approach does not support 3D images, and therefore, they focus only on plate recognition, not detecting and identifying fraud scenarios.

A recent paper (Patel et al., 2021) introduced a semantic representation of suspicious vehicle features used to detect malicious activities. The automatic tracking systems using ontologies is the purpose of paper (Greco, Ritrovato & Vento, 2017). They define several SWRL rules to track cars and walking persons meanwhile tagging them as suspects or not. However, their ontology does not consider records management, different used device specifications, or the involved actors in the tracking event.

SanMiguel, Martínez & García (2009) proposed a semantic representation of prior knowledge related to video events analysis. Their ontology mainly models the domain knowledge and system knowledge. Their data representation model is extended by SanMiguel & Martinez (2013) to include more details about context knowledge, the scene, and user preferences.

Francois et al. (2005) proposed the Video Event Representation Language(VERL) to describe video events and the Video Event Markup Language (VEML) to annotate event instances. Their semantic representation is considered among the first dealing with video events modeling. Recently, automated driving vehicles have received an increased focus due to the large auto-cars spreading. Elgharbawy et al. (2019) and Li, Tao & Wotawa (2020) proposed similar approaches using ontology to test several generated scenarios to validate required functional safety. Indeed, the effort in Elgharbawy et al. (2019) uses a data mining technique to extract representation scenarios witnessed in real-world traffic from the ontology-based database.

Table 9 depicts the required features to solve the challenges posed by this paper and compares the relevant existing efforts in terms of their satisfaction with those features or not. Briefly, those comparing features are:

• Extensibility: the ability of the proposed approach to incorporate new requirements such as new technologies or linked with other similar vehicle tracking systems.
• Forensics consideration: does the proposed approach considers forensics requirements attributes.
• Scalability: do the system management and associated data model able to deal with a significant increase of vehicles or captured data.
• Interoperability: could the proposed approach be easily integrated with already existing systems.
• Automatic reasoning: do the proposed approach implement and enhance the automatic reasoning to detect and deduce committed frauds.
• Multiple fraud types: does the proposed approach model several fraud scenarios or jnot.

According to the depicted results, none of the relevant related efforts to this paper has fully responded to the required features. More specifically, none of them merges forensics requirements into their proposed ontology. Thus, any evidence is subject to admissibility issues. This paper, however, identifies relevant suspicious patterns about vehicle frauds in a forensically sound manner. It is achieved by using a scalable, secure management framework and an extensible forensics-aware auto-reasoning data model.

**Table 9:** Comparison of the relevant identified research efforts.

References	Extensibi- lity	Forensics consideration	Scalability	Interoper- ability	Automatic reasoning	Multiple fraud types
mostfa2019design			✓			
patel2021video	✓					✓
greco2017advanced	✓				✓	✓
sanmiguel2009ontology	✓				✓	✓
francois2005verl	✓				✓	✓
elgharbawy2019ontology	✓				✓	✓
li2020ontology	✓					✓
FCVRO	✓	✓	✓	✓	✓	✓

This paper also uses standards to build the data model, which provides high interoperability, enabling easier integration. The scalability is achieved through the cluster-based distributed management framework that offers onsite data processing and then deep processing through the cloud-based solution. The extensibility, automatic reasoning, and definition of multiple fraud scenarios are ensured by adopting an ontology-based data model and using reasoning engines to infer new knowledge triggered by the defined SWRL rules and SPARQL queries.

Since forensics legislation and understanding are different for each country, the integrated forensics requirement and implemented rules must be adapted according to the country’s laws. It requires experts to integrate them, although the ontology uses standards to modify it easily. Also, it is essential today to consider including IoT requirements, once used with checkpoint managements, within the ontology to keep with the world’s tendencies towards smart cities such as NEOM, the newly established fast-growing smart city in Saudi Arabia (https://www.neom.com/en-us). More specifically, the ontology may incorporate the intelligent routing of self-driving vehicles (Celsi et al., 2017). This feature will enable the ontology to respond to future requirements while keeping the admissibility of the records.

## Conclusion

The increased use of cameras to detect roads frauds raises several issues associated with significant data communicated between cameras and processing units, scalability and real-time detection, automation of the data search for important information, and data admissibility preservation.

To address these gaps, this paper defines the different checkpoint control system specifications and design requirements. Then, it proposes a new architectural framework that adheres to the system specification. Besides, this study provides a new checkpoint’s vehicle recognition ontology to identify suspicious vehicles, their tracking, and search from the associated events. Aside from the proposed cluster-based multi-checkpoints management system, the main contribution of this study is the forensic-oriented design of the ontology to respond to all court requirements regarding the gathered evidence admissibility. The paper uses standards during the establishment of the new data model to improve and ensure its easy integration with already existing similar systems.

As future work, the plan is to extend the ontology to include prior knowledge of the scene, which helps in improving tracking performance. Furthermore, anonymization techniques (Akremi & Rouached, 2021; El Ouazzani & El Bakkali, 2020) will be used to protect the ontology data privacy without decreasing the real-time detection of suspicious vehicles and system control scalability.

## Supplemental Information

10.7717/peerj-cs.841/supp-1 Supplemental Information 1 HTML documentation of the proposed ontology An auto generated protege’s documentation of the proposed ontology.

10.7717/peerj-cs.841/supp-2 Supplemental Information 2 The ontology source file An ontology of forensics-aware checkpoint’s vehicle inspection.

## Additional Information and Declarations

### Data Availability

The following information was supplied regarding data availability: The raw data is available in the Supplemental Files.
