# Supplementary material for: A forensic-driven data model for automatic vehicles events analysis
Source: PeerJ Comput Sci. 2022 Jan 5;8:e841. doi: 10.7717/peerj-cs.841 (PMC8771793; doi:10.7717/peerj-cs.841)
Supplement: Supplemental Information 1 — An auto generated protege’s documentation of the proposed ontology. [file peerj-cs-08-841-s001.zip › Vro_Html/index-all.html]

Ontology Browser


Ontologies
Classes
Object Properties
Data Properties
Annotation Properties
Individuals
Datatypes
Clouds

## Entities (126)

- owl:Thing
- age
- alternativeOf
- Assessment
- associatedWith
- bodyTypeEnum
- brandEnum
- capturedBy
- car1
- Chekpoint
- comment
- connects
- Contact
- contactID
- contactName
- contactType
- contactTypeEnum
- contains
- Country
- creationTime
- currency
- description
- detectTime
- deviceDescription
- deviceID
- deviceName
- deviceType
- deviceTypeEnum
- Distance
- drivedBy
- duration
- endTime
- Eric
- estimatedby
- EvaluatedBy
- Event
- expectedfrom
- Fraud
- Fraud01
- fraudID
- fraudType
- fraudTypeEnum
- gender
- generatedBy
- handledBy
- Hardware
- has
- hasBodyType
- hasBrand
- hasChasisNumber
- hasColor
- hasCoordinates
- hasDistance
- hasModel
- hasPassengersNumber
- hasPlateNumber
- HasSpeedLimit
- hasType
- hasTypeEnum
- identifiedBy
- Impact
- Incident
- incidentID
- incidentType
- incidentTypeEnum
- includes
- involves
- isRuleEnabled
- isStolen
- label
- locatedIn
- mayBe
- MonetaryImpact
- Network
- networkSender
- networkType
- networkTypeEnum
- netwrokReceiver
- NorthCheckpoint
- owl:Nothing
- performedBy
- PlainLiteral
- pointOfHit
- pointOfHitEnum
- rdf:PlainLiteral
- rdfs:comment
- rdfs:label
- Record
- recordID
- recordName
- recordSize
- Reference
- RelatedActivity
- relates
- runs
- runsOn
- secID
- secType
- secTypeEnum
- Security
- severity
- sexTypeEnum
- softID
- softName
- softType
- softTypeEnum
- Software
- specUse
- speed
- speedLimitEnum
- storedBy
- storedIn
- TechnicalImpact
- TimeImpact
- transmittedVia
- uses
- Vehicle
- vehicleModelEnum
- vehicleStatus
- vehicleStatusEnum
- versionInfo
- xsd:boolean
- xsd:dateTime
- xsd:float
- xsd:integer
- xsd:string

OWL HTML inside
