# Supplementary material for: A forensic-driven data model for automatic vehicles events analysis
Source: PeerJ Comput Sci. 2022 Jan 5;8:e841. doi: 10.7717/peerj-cs.841 (PMC8771793; doi:10.7717/peerj-cs.841)
Supplement: Supplemental Information 1 — An auto generated protege’s documentation of the proposed ontology. [file peerj-cs-08-841-s001.zip › Vro_Html/annotationproperties/index.html]

## All Annotationproperties (6)

- comment
- isRuleEnabled
- label
- rdfs:comment
- rdfs:label
- versionInfo
