# Supplementary material for: A forensic-driven data model for automatic vehicles events analysis
Source: PeerJ Comput Sci. 2022 Jan 5;8:e841. doi: 10.7717/peerj-cs.841 (PMC8771793; doi:10.7717/peerj-cs.841)
Supplement: Supplemental Information 1 — An auto generated protege’s documentation of the proposed ontology. [file peerj-cs-08-841-s001.zip › Vro_Html/ontologies/vro___-1348188527.html]

Ontology Browser


Ontologies
Classes
Object Properties
Data Properties
Annotation Properties
Individuals
Datatypes
Clouds

## vro

### http://www.expertisebeat.com/vro

### Loaded from file:/C:/Users/User/Desktop/vro.owl

#### Annotations (1)

- rdfs:comment "This is an ontology about Vehicle detection, events analysis, and considering forensics requirements" @en

#### References

- Classes (22)
- Object Properties (29)
- Data Properties (45)
- Annotation Properties (6)
- Individuals (4)
- Datatypes (25)

OWL HTML inside
