# Supplementary material for: A forensic-driven data model for automatic vehicles events analysis
Source: PeerJ Comput Sci. 2022 Jan 5;8:e841. doi: 10.7717/peerj-cs.841 (PMC8771793; doi:10.7717/peerj-cs.841)
Supplement: Supplemental Information 1 — An auto generated protege’s documentation of the proposed ontology. [file peerj-cs-08-841-s001.zip › Vro_Html/objectproperties/index-vro.html]

Ontology Browser


Ontologies
Classes
Object Properties
Data Properties
Annotation Properties
Individuals
Datatypes
Clouds

## vro: objectproperties (27)

- alternativeOf
- associatedWith
- capturedBy
- connects
- contains
- drivedBy
- estimatedby
- EvaluatedBy
- expectedfrom
- generatedBy
- handledBy
- has
- hasDistance
- identifiedBy
- includes
- involves
- isStolen
- locatedIn
- mayBe
- performedBy
- relates
- runs
- runsOn
- storedBy
- storedIn
- transmittedVia
- uses

OWL HTML inside
