# Supplementary material for: A forensic-driven data model for automatic vehicles events analysis
Source: PeerJ Comput Sci. 2022 Jan 5;8:e841. doi: 10.7717/peerj-cs.841 (PMC8771793; doi:10.7717/peerj-cs.841)
Supplement: Supplemental Information 1 — An auto generated protege’s documentation of the proposed ontology. [file peerj-cs-08-841-s001.zip › Vro_Html/objectproperties/connects___-749035401.html]

Ontology Browser


Ontologies
Classes
Object Properties
Data Properties
Annotation Properties
Individuals
Datatypes
Clouds

## Object Property: connects

#### Domains (1)

- Network

#### Ranges (2)

- Chekpoint
- Hardware

OWL HTML inside
