# Supplementary material for: A forensic-driven data model for automatic vehicles events analysis
Source: PeerJ Comput Sci. 2022 Jan 5;8:e841. doi: 10.7717/peerj-cs.841 (PMC8771793; doi:10.7717/peerj-cs.841)
Supplement: Supplemental Information 1 — An auto generated protege’s documentation of the proposed ontology. [file peerj-cs-08-841-s001.zip › Vro_Html/individuals/Eric___-173993387.html]

Ontology Browser


Ontologies
Classes
Object Properties
Data Properties
Annotation Properties
Individuals
Datatypes
Clouds

## Individual: Eric

#### Types (1)

- Contact

#### age

- 32

#### contactID

- "1001001"(xsd:string)

#### contactName

- "Eric"(xsd:string)

#### contactType

- "Driver"

#### gender

- "Male"

#### Usage (1)

- Fraud01 involves Eric

OWL HTML inside
