# Supplementary material for: A forensic-driven data model for automatic vehicles events analysis
Source: PeerJ Comput Sci. 2022 Jan 5;8:e841. doi: 10.7717/peerj-cs.841 (PMC8771793; doi:10.7717/peerj-cs.841)
Supplement: Supplemental Information 1 — An auto generated protege’s documentation of the proposed ontology. [file peerj-cs-08-841-s001.zip › Vro_Html/individuals/car1___-173115765.html]

Ontology Browser


Ontologies
Classes
Object Properties
Data Properties
Annotation Properties
Individuals
Datatypes
Clouds

## Individual: car1

#### Types (3)

- Fraud
- Vehicle
- fraudType some fraudTypeEnum

#### age

- 32

#### contactID

- "1001001"(xsd:string)

#### contactName

- "Eric"(xsd:string)

#### contactType

- "Driver"

#### gender

- "Male"

#### fraudID

- "F01"

#### fraudType

- "Stolen"

#### involves

- Eric

#### hasCoordinates

- "123,456"(xsd:string)

#### HasSpeedLimit

- "40"(xsd:string)

#### hasBodyType

- "Sedan"

#### hasBrand

- "Toyota"

#### hasChasisNumber

- "1234VF567XR800"

#### hasColor

- "Blue"

#### hasModel

- 2021

#### hasPassengersNumber

- 5

#### hasPlateNumber

- "5694 SA 23"

#### hasType

- "nauto"

#### locatedIn

- NorthCheckpoint

#### vehicleStatus

- "Stolen"

OWL HTML inside
