# Supplementary material for: A forensic-driven data model for automatic vehicles events analysis
Source: PeerJ Comput Sci. 2022 Jan 5;8:e841. doi: 10.7717/peerj-cs.841 (PMC8771793; doi:10.7717/peerj-cs.841)
Supplement: Supplemental Information 1 — An auto generated protege’s documentation of the proposed ontology. [file peerj-cs-08-841-s001.zip › Vro_Html/classes/Hardware___5391798.html]

Ontology Browser


Ontologies
Classes
Object Properties
Data Properties
Annotation Properties
Individuals
Datatypes
Clouds

## Class: Hardware

#### Annotations (1)

- rdfs:comment "Hardware (encompasses the different hardware elements such as cameras, routers, computers, etc.)"(xsd:string)

#### Superclasses (1)

- owl:Thing

#### Usage (14)

- runs Domain Hardware
- capturedBy Range Hardware
- connects Range Hardware
- contains Range Hardware
- generatedBy Range Hardware
- mayBe Range Hardware
- runsOn Range Hardware
- storedBy Range Hardware
- storedIn Range Hardware
- uses Range Hardware
- deviceDescription Domain Hardware
- deviceID Domain Hardware
- deviceName Domain Hardware
- deviceType Domain Hardware

OWL HTML inside
