# Supplementary material for: A forensic-driven data model for automatic vehicles events analysis
Source: PeerJ Comput Sci. 2022 Jan 5;8:e841. doi: 10.7717/peerj-cs.841 (PMC8771793; doi:10.7717/peerj-cs.841)
Supplement: Supplemental Information 1 — An auto generated protege’s documentation of the proposed ontology. [file peerj-cs-08-841-s001.zip › Vro_Html/classes/Record___-2027203553.html]

Ontology Browser


Ontologies
Classes
Object Properties
Data Properties
Annotation Properties
Individuals
Datatypes
Clouds

## Class: Record

#### Annotations (1)

- rdfs:comment "Within the record module, the forensics requirements in the ontology design are incorporated acheived through security techniques. The record module mainly deals with the forensically sound processing and preservation of generated files and data for further use."(xsd:string)

#### Superclasses (1)

- owl:Thing

#### Usage (9)

- identifiedBy Domain Record
- storedBy Domain Record
- storedIn Domain Record
- transmittedVia Domain Record
- creationTime Domain Record
- description Domain Record
- recordID Domain Record
- recordName Domain Record
- recordSize Domain Record

OWL HTML inside
