# Supplementary material for: A forensic-driven data model for automatic vehicles events analysis
Source: PeerJ Comput Sci. 2022 Jan 5;8:e841. doi: 10.7717/peerj-cs.841 (PMC8771793; doi:10.7717/peerj-cs.841)
Supplement: Supplemental Information 1 — An auto generated protege’s documentation of the proposed ontology. [file peerj-cs-08-841-s001.zip › Vro_Html/classes/Contact___-1854949458.html]

Ontology Browser


Ontologies
Classes
Object Properties
Data Properties
Annotation Properties
Individuals
Datatypes
Clouds

## Class: Contact

#### Annotations (1)

- rdfs:comment "The purpose of the Contact module is to model all human beings in contact with the recognition process. Contact may be the police agents, system administrators, vehicle drivers, and any possible person that may impact the final recognition process."(xsd:string)

#### Superclasses (1)

- owl:Thing

#### Members (1)

Eric

#### Usage (15)

- associatedWith Domain Contact
- associatedWith Range Contact
- contains Range Contact
- drivedBy Range Contact
- generatedBy Range Contact
- handledBy Range Contact
- involves Range Contact
- performedBy Range Contact
- storedBy Range Contact
- age Domain Contact
- contactID Domain Contact
- contactName Domain Contact
- contactType Domain Contact
- gender Domain Contact

OWL HTML inside
