# Supplementary material for: A forensic-driven data model for automatic vehicles events analysis
Source: PeerJ Comput Sci. 2022 Jan 5;8:e841. doi: 10.7717/peerj-cs.841 (PMC8771793; doi:10.7717/peerj-cs.841)
Supplement: Supplemental Information 1 — An auto generated protege’s documentation of the proposed ontology. [file peerj-cs-08-841-s001.zip › Vro_Html/classes/Fraud___-108022286.html]

Ontology Browser


Ontologies
Classes
Object Properties
Data Properties
Annotation Properties
Individuals
Datatypes
Clouds

## Class: Fraud

#### Annotations (1)

- rdfs:comment "The fraud module describes the various possible frauds the system aims to identify. It is connected only with the event class to determine the event fraud type."(xsd:string)

#### Superclasses (1)

- owl:Thing

#### Members (2)

car1,
Fraud01

#### Usage (7)

- involves Domain Fraud
- description Domain Fraud
- fraudID Domain Fraud
- fraudType Domain Fraud
- pointOfHit Domain Fraud
- speed Domain Fraud

OWL HTML inside
