# Supplementary material for: A forensic-driven data model for automatic vehicles events analysis
Source: PeerJ Comput Sci. 2022 Jan 5;8:e841. doi: 10.7717/peerj-cs.841 (PMC8771793; doi:10.7717/peerj-cs.841)
Supplement: Supplemental Information 1 — An auto generated protege’s documentation of the proposed ontology. [file peerj-cs-08-841-s001.zip › Vro_Html/classes/Assessment___-358281584.html]

Ontology Browser


Ontologies
Classes
Object Properties
Data Properties
Annotation Properties
Individuals
Datatypes
Clouds

## Class: Assessment

#### Superclasses (1)

- owl:Thing

#### Usage (2)

- estimatedby Domain Assessment
- EvaluatedBy Range Assessment

OWL HTML inside
