# Supplementary material for: A forensic-driven data model for automatic vehicles events analysis
Source: PeerJ Comput Sci. 2022 Jan 5;8:e841. doi: 10.7717/peerj-cs.841 (PMC8771793; doi:10.7717/peerj-cs.841)
Supplement: Supplemental Information 1 — An auto generated protege’s documentation of the proposed ontology. [file peerj-cs-08-841-s001.zip › Vro_Html/classes/index-vro.html]

Ontology Browser


Ontologies
Classes
Object Properties
Data Properties
Annotation Properties
Individuals
Datatypes
Clouds

## vro: classes (22)

- owl:Thing
- Assessment
- Chekpoint
- Contact
- Country
- Distance
- Event
- Fraud
- Hardware
- Impact
- Incident
- MonetaryImpact
- Network
- owl:Nothing
- Record
- Reference
- RelatedActivity
- Security
- Software
- TechnicalImpact
- TimeImpact
- Vehicle

OWL HTML inside
