# Supplementary material for: A forensic-driven data model for automatic vehicles events analysis
Source: PeerJ Comput Sci. 2022 Jan 5;8:e841. doi: 10.7717/peerj-cs.841 (PMC8771793; doi:10.7717/peerj-cs.841)
Supplement: Supplemental Information 1 — An auto generated protege’s documentation of the proposed ontology. [file peerj-cs-08-841-s001.zip › Vro_Html/classes/Vehicle___1830560442.html]

Ontology Browser


Ontologies
Classes
Object Properties
Data Properties
Annotation Properties
Individuals
Datatypes
Clouds

## Class: Vehicle

#### Superclasses (1)

- owl:Thing

#### Members (1)

car1

#### Usage (13)

- drivedBy Domain Vehicle
- isStolen Domain Vehicle
- locatedIn Domain Vehicle
- hasBodyType Domain Vehicle
- hasBrand Domain Vehicle
- hasChasisNumber Domain Vehicle
- hasColor Domain Vehicle
- hasModel Domain Vehicle
- hasPassengersNumber Domain Vehicle
- hasPlateNumber Domain Vehicle
- hasType Domain Vehicle
- vehicleStatus Domain Vehicle

OWL HTML inside
