# Supplementary material for: A forensic-driven data model for automatic vehicles events analysis
Source: PeerJ Comput Sci. 2022 Jan 5;8:e841. doi: 10.7717/peerj-cs.841 (PMC8771793; doi:10.7717/peerj-cs.841)
Supplement: Supplemental Information 1 — An auto generated protege’s documentation of the proposed ontology. [file peerj-cs-08-841-s001.zip › Vro_Html/classes/Event___-108823000.html]

Ontology Browser


Ontologies
Classes
Object Properties
Data Properties
Annotation Properties
Individuals
Datatypes
Clouds

## Class: Event

#### Annotations (1)

- rdfs:comment "An event is the smallest complete task that occurred by an active part. The incident describes all actions and events within checkpoints and/or within the intermediate systems and tools. Each incident may have one or several events."(xsd:string)

#### Superclasses (1)

- owl:Thing

#### Members (1)

Fraud01

#### Usage (9)

- capturedBy Domain Event
- generatedBy Domain Event
- handledBy Domain Event
- involves Domain Event
- performedBy Domain Event
- includes Range Event
- description Domain Event
- detectTime Domain Event
- endTime Domain Event

OWL HTML inside
