# Supplementary material for: A forensic-driven data model for automatic vehicles events analysis
Source: PeerJ Comput Sci. 2022 Jan 5;8:e841. doi: 10.7717/peerj-cs.841 (PMC8771793; doi:10.7717/peerj-cs.841)
Supplement: Supplemental Information 1 — An auto generated protege’s documentation of the proposed ontology. [file peerj-cs-08-841-s001.zip › Vro_Html/classes/Security___837605134.html]

Ontology Browser


Ontologies
Classes
Object Properties
Data Properties
Annotation Properties
Individuals
Datatypes
Clouds

## Class: Security

#### Annotations (1)

- rdfs:comment "The Security module's purpose is to provide all required security provisions for other modules including software or hardware tools. Aside from protecting the system from malicious and hacker penetration, this module enables forensically sound record processing and preservation, such as providing integrity techniques (MD5, Sha1, etc.)."(xsd:string)

#### Superclasses (1)

- owl:Thing

#### Usage (5)

- mayBe Domain Security
- uses Range Security
- description Domain Security
- secID Domain Security
- secType Domain Security

OWL HTML inside
