# Supplementary material for: A forensic-driven data model for automatic vehicles events analysis
Source: PeerJ Comput Sci. 2022 Jan 5;8:e841. doi: 10.7717/peerj-cs.841 (PMC8771793; doi:10.7717/peerj-cs.841)
Supplement: Supplemental Information 1 — An auto generated protege’s documentation of the proposed ontology. [file peerj-cs-08-841-s001.zip › Vro_Html/classes/Chekpoint___-1834122189.html]

Ontology Browser


Ontologies
Classes
Object Properties
Data Properties
Annotation Properties
Individuals
Datatypes
Clouds

## Class: Chekpoint

#### Annotations (1)

- rdfs:comment "The vehicle module encompasses all attributes that distinguish a vehicle from another. It determines whether the car is self-driving (auto) or not (nauto). Also, it determines the legal status of the vehicle. This module communicates with the Contact module since each vehicle has a driver and eventual passengers and checkpoint module where the vehicle is located and is subject to the recognition process."(xsd:string)

#### Superclasses (1)

- owl:Thing

#### Members (1)

NorthCheckpoint

#### Usage (10)

- contains Domain Chekpoint
- has Domain Chekpoint
- hasDistance Domain Chekpoint
- connects Range Chekpoint
- expectedfrom Range Chekpoint
- hasDistance Range Chekpoint
- locatedIn Range Chekpoint
- HasSpeedLimit Domain Chekpoint
- duration Domain Chekpoint
- hasCoordinates Domain Chekpoint

OWL HTML inside
