# Supplementary material for: A forensic-driven data model for automatic vehicles events analysis
Source: PeerJ Comput Sci. 2022 Jan 5;8:e841. doi: 10.7717/peerj-cs.841 (PMC8771793; doi:10.7717/peerj-cs.841)
Supplement: Supplemental Information 1 — An auto generated protege’s documentation of the proposed ontology. [file peerj-cs-08-841-s001.zip › Vro_Html/classes/Incident___-24533856.html]

Ontology Browser


Ontologies
Classes
Object Properties
Data Properties
Annotation Properties
Individuals
Datatypes
Clouds

## Class: Incident

#### Annotations (1)

- rdfs:comment "Each incident may have one or several events. An event is the smallest complete task that occurred by an active part. The incident describes all actions and events within checkpoints and/or within the intermediate systems and tools. Thus, an incident may cover several events generated from different checkpoints or/and medians. An incident may be internal (caused by internal contact) or external (caused by external contact such as a new suspected vehicle)."(xsd:string)

#### Superclasses (1)

- owl:Thing

#### Usage (10)

- EvaluatedBy Domain Incident
- alternativeOf Domain Incident
- includes Domain Incident
- alternativeOf Range Incident
- relates Range Incident
- description Domain Incident
- detectTime Domain Incident
- endTime Domain Incident
- incidentID Domain Incident
- incidentType Domain Incident

OWL HTML inside
