# Supplementary material for: A forensic-driven data model for automatic vehicles events analysis
Source: PeerJ Comput Sci. 2022 Jan 5;8:e841. doi: 10.7717/peerj-cs.841 (PMC8771793; doi:10.7717/peerj-cs.841)
Supplement: Supplemental Information 1 — An auto generated protege’s documentation of the proposed ontology. [file peerj-cs-08-841-s001.zip › Vro_Html/datatypes/vehicleModelEnum___585115052.html]

Ontology Browser


Ontologies
Classes
Object Properties
Data Properties
Annotation Properties
Individuals
Datatypes
Clouds

## Datatype: vehicleModelEnum

#### Datatype Definitions (1)

- {"2000", "2001", "2002", "2003", "2004", "2005", "2006", "2007", "2008", "2009", "2010", "2011", "2012", "2013", "2014", "2015", "2016", "2017", "2018", "2019", "2020-", "2021"}

#### Usage (1)

- vehicleModelEnum EquivalentTo {"2000", "2001", "2002", "2003", "2004", "2005", "2006", "2007", "2008", "2009", "2010", "2011", "2012", "2013", "2014", "2015", "2016", "2017", "2018", "2019", "2020-", "2021"}

OWL HTML inside
