# Supplementary material for: A forensic-driven data model for automatic vehicles events analysis
Source: PeerJ Comput Sci. 2022 Jan 5;8:e841. doi: 10.7717/peerj-cs.841 (PMC8771793; doi:10.7717/peerj-cs.841)
Supplement: Supplemental Information 1 — An auto generated protege’s documentation of the proposed ontology. [file peerj-cs-08-841-s001.zip › Vro_Html/datatypes/index.html]

## All Datatypes (24)

- bodyTypeEnum
- brandEnum
- contactTypeEnum
- deviceTypeEnum
- fraudTypeEnum
- hasTypeEnum
- incidentTypeEnum
- networkTypeEnum
- PlainLiteral
- pointOfHitEnum
- rdf:PlainLiteral
- secTypeEnum
- severity
- sexTypeEnum
- softTypeEnum
- specUse
- speedLimitEnum
- vehicleModelEnum
- vehicleStatusEnum
- xsd:boolean
- xsd:dateTime
- xsd:float
- xsd:integer
- xsd:string
