# Supplementary material for: A forensic-driven data model for automatic vehicles events analysis
Source: PeerJ Comput Sci. 2022 Jan 5;8:e841. doi: 10.7717/peerj-cs.841 (PMC8771793; doi:10.7717/peerj-cs.841)
Supplement: Supplemental Information 1 — An auto generated protege’s documentation of the proposed ontology. [file peerj-cs-08-841-s001.zip › Vro_Html/datatypes/PlainLiteral___-1770072891.html]

Ontology Browser


Ontologies
Classes
Object Properties
Data Properties
Annotation Properties
Individuals
Datatypes
Clouds

## Datatype: rdf:PlainLiteral

#### Usage (43)

- Eric contactType "Driver"
- Eric gender "Male"
- Fraud01 fraudID "F01"
- Fraud01 fraudType "Stolen"
- car1 hasBodyType "Sedan"
- car1 hasBrand "Toyota"
- car1 hasChasisNumber "1234VF567XR800"
- car1 hasColor "Blue"
- car1 hasPlateNumber "5694 SA 23"
- car1 hasType "nauto"
- car1 vehicleStatus "Stolen"
- HasSpeedLimit Range {"100", "120", "40", "60", "70", "80", "90"}
- contactType Range {"Administrator", "Driver", "Investigator", "Passenger", "Security agent"}
- deviceType Range {"Administrator", "Driver", "Investigator", "Passenger", "Security agent"}
- fraudType Range {"Hit", "Licence plate fraud", "Possession of prohibited items", "Speed fraud", "Stolen", "Theft fraud", "Transportation of offenders"}
- hasBodyType Range {"Fan", "Pick-Up", "Sedan"}
- hasBrand Range {"Abarth", "Alfa Romeo", "Aston Martin", "Audi", "BMW", "Bentley", "Bugatti", "Cadillac", "Chevrolet", "Chrysler", "Citroën", "DS", "Dacia", "Daewoo", "Daihatsu", "Dodge", "Donkervoort", "Ferrari", "Fiat", "Fisker", "Ford", "Honda", "Hummer", "Hyundai", "Infiniti", "Iveco", "Jaguar", "Jeep", "KTM", "Kia", "Lada", "Lamborghini", "Lancia", "Land Rover", "Landwind", "Lexus", "Lotus", "MG", "Maserati", "Maybach", "Mazda", "McLaren", "Mercedes-Benz", "Mini", "Mitsubishi", "Morgan", "Nissan", "Opel", "Peugeot", "Porsche", "Renault", "Rolls-Royce", "Rover", "Saab", "Seat", "Skoda", "Smart", "SsangYong", "Subaru", "Suzuki", "Tesla", "Toyota", "Volkswagen", "Volvo"}
- hasType Range {"auto", "nauto"}
- incidentType Range {"both", "external", "internal"}
- networkType Range {"Wire", "WireLess"}
- pointOfHit Range {"Back hit", "Front hit", "Left side hit", "Right side hit"}
- secType Range {"Compensating", "Corrective", "Detective", "Deterrent", "Directive", "Preventive", "Recovery"}
- softType Range {"Cameras Management", "Network", "Operating Systems", "Security"}
- vehicleStatus Range {"Nothing", "Stolen", "Wanted"}
- incidentTypeEnum comment "determines the causer of the incident ether internal (example security agent), external(example citizen"
- secTypeEnum rdfs:comment "Security provisions categories" @en
- bodyTypeEnum EquivalentTo {"Fan", "Pick-Up", "Sedan"}
- brandEnum EquivalentTo {"Abarth", "Alfa Romeo", "Aston Martin", "Audi", "BMW", "Bentley", "Bugatti", "Cadillac", "Chevrolet", "Chrysler", "Citroën", "DS", "Dacia", "Daewoo", "Daihatsu", "Dodge", "Donkervoort", "Ferrari", "Fiat", "Fisker", "Ford", "Honda", "Hummer", "Hyundai", "Infiniti", "Iveco", "Jaguar", "Jeep", "KTM", "Kia", "Lada", "Lamborghini", "Lancia", "Land Rover", "Landwind", "Lexus", "Lotus", "MG", "Maserati", "Maybach", "Mazda", "McLaren", "Mercedes-Benz", "Mini", "Mitsubishi", "Morgan", "Nissan", "Opel", "Peugeot", "Porsche", "Renault", "Rolls-Royce", "Rover", "Saab", "Seat", "Skoda", "Smart", "SsangYong", "Subaru", "Suzuki", "Tesla", "Toyota", "Volkswagen", "Volvo"}
- contactTypeEnum EquivalentTo {"Administrator", "Driver", "Investigator", "Passenger", "Security agent"}
- deviceTypeEnum EquivalentTo {"3DCamera", "Camera", "CloudServer", "Computer", "LaserCamera", "Mobile", "Router", "Server"}
- fraudTypeEnum EquivalentTo {"Hit", "Licence plate fraud", "Possession of prohibited items", "Speed fraud", "Stolen", "Theft fraud", "Transportation of offenders"}
- hasTypeEnum EquivalentTo {"auto", "nauto"}
- incidentTypeEnum EquivalentTo {"both", "external", "internal"}
- networkTypeEnum EquivalentTo {"Wire", "WireLess"}
- pointOfHitEnum EquivalentTo {"Back hit", "Front hit", "Left side hit", "Right side hit"}
- secTypeEnum EquivalentTo {"Compensating", "Corrective", "Detective", "Deterrent", "Directive", "Preventive", "Recovery"}
- severity EquivalentTo {"High", "Low", "Meduim"}
- sexTypeEnum EquivalentTo {"Female", "Male"}
- softTypeEnum EquivalentTo {"Cameras Management", "Network", "Operating Systems", "Security"}
- specUse EquivalentTo {"Commercial Transportation", "Disabled Transportation", "Food Transprotation", "Government Transprotation", "Patient Transportation"}
- speedLimitEnum EquivalentTo {"100", "120", "40", "60", "70", "80", "90"}
- vehicleModelEnum EquivalentTo {"2000", "2001", "2002", "2003", "2004", "2005", "2006", "2007", "2008", "2009", "2010", "2011", "2012", "2013", "2014", "2015", "2016", "2017", "2018", "2019", "2020-", "2021"}
- vehicleStatusEnum EquivalentTo {"Nothing", "Wanted"}

OWL HTML inside
