# Supplementary material for: A forensic-driven data model for automatic vehicles events analysis
Source: PeerJ Comput Sci. 2022 Jan 5;8:e841. doi: 10.7717/peerj-cs.841 (PMC8771793; doi:10.7717/peerj-cs.841)
Supplement: Supplemental Information 1 — An auto generated protege’s documentation of the proposed ontology. [file peerj-cs-08-841-s001.zip › Vro_Html/datatypes/string___799955032.html]

Ontology Browser


Ontologies
Classes
Object Properties
Data Properties
Annotation Properties
Individuals
Datatypes
Clouds

## Datatype: xsd:string

#### Usage (35)

- Eric contactID "1001001"(xsd:string)
- Eric contactName "Eric"(xsd:string)
- NorthCheckpoint HasSpeedLimit "40"(xsd:string)
- NorthCheckpoint hasCoordinates "123,456"(xsd:string)
- contactID Range xsd:string
- contactName Range xsd:string
- currency Range xsd:string
- description Range xsd:string
- deviceDescription Range xsd:string
- deviceID Range xsd:string
- deviceName Range xsd:string
- fraudID Range xsd:string
- hasChasisNumber Range xsd:string
- hasColor Range xsd:string
- hasCoordinates Range xsd:string
- hasPlateNumber Range xsd:string
- incidentID Range xsd:string
- networkSender Range xsd:string
- netwrokReceiver Range xsd:string
- recordID Range xsd:string
- recordName Range xsd:string
- secID Range xsd:string
- softID Range xsd:string
- softName Range xsd:string
- Chekpoint rdfs:comment "The vehicle module encompasses all attributes that distinguish a vehicle from another. It determines whether the car is self-driving (auto) or not (nauto). Also, it determines the legal status of the vehicle. This module communicates with the Contact module since each vehicle has a driver and eventual passengers and checkpoint module where the vehicle is located and is subject to the recognition process."(xsd:string)
- Contact rdfs:comment "The purpose of the Contact module is to model all human beings in contact with the recognition process. Contact may be the police agents, system administrators, vehicle drivers, and any possible person that may impact the final recognition process."(xsd:string)
- Event rdfs:comment "An event is the smallest complete task that occurred by an active part. The incident describes all actions and events within checkpoints and/or within the intermediate systems and tools. Each incident may have one or several events."(xsd:string)
- Fraud rdfs:comment "The fraud module describes the various possible frauds the system aims to identify. It is connected only with the event class to determine the event fraud type."(xsd:string)
- Hardware rdfs:comment "Hardware (encompasses the different hardware elements such as cameras, routers, computers, etc.)"(xsd:string)
- Incident rdfs:comment "Each incident may have one or several events. An event is the smallest complete task that occurred by an active part. The incident describes all actions and events within checkpoints and/or within the intermediate systems and tools. Thus, an incident may cover several events generated from different checkpoints or/and medians. An incident may be internal (caused by internal contact) or external (caused by external contact such as a new suspected vehicle)."(xsd:string)
- Network rdfs:comment "network (defines the various used network interfaces and the different connections as well as the used technologies)"(xsd:string)
- Record rdfs:comment "Within the record module, the forensics requirements in the ontology design are incorporated acheived through security techniques. The record module mainly deals with the forensically sound processing and preservation of generated files and data for further use."(xsd:string)
- Security rdfs:comment "The Security module's purpose is to provide all required security provisions for other modules including software or hardware tools. Aside from protecting the system from malicious and hacker penetration, this module enables forensically sound record processing and preservation, such as providing integrity techniques (MD5, Sha1, etc.)."(xsd:string)
- Software rdfs:comment "software (includes all software requirements such as recognition and transmission methods, soft security tools)"(xsd:string)

OWL HTML inside
