# Supplementary material for: A forensic-driven data model for automatic vehicles events analysis
Source: PeerJ Comput Sci. 2022 Jan 5;8:e841. doi: 10.7717/peerj-cs.841 (PMC8771793; doi:10.7717/peerj-cs.841)
Supplement: Supplemental Information 1 — An auto generated protege’s documentation of the proposed ontology. [file peerj-cs-08-841-s001.zip › Vro_Html/dataproperties/index.html]

## All Dataproperties (43)

- age
- contactID
- contactName
- contactType
- creationTime
- currency
- description
- detectTime
- deviceDescription
- deviceID
- deviceName
- deviceType
- duration
- endTime
- fraudID
- fraudType
- gender
- hasBodyType
- hasBrand
- hasChasisNumber
- hasColor
- hasCoordinates
- hasModel
- hasPassengersNumber
- hasPlateNumber
- HasSpeedLimit
- hasType
- incidentID
- incidentType
- networkSender
- networkType
- netwrokReceiver
- pointOfHit
- recordID
- recordName
- recordSize
- secID
- secType
- softID
- softName
- softType
- speed
- vehicleStatus
