# Supplementary material for: A forensic-driven data model for automatic vehicles events analysis
Source: PeerJ Comput Sci. 2022 Jan 5;8:e841. doi: 10.7717/peerj-cs.841 (PMC8771793; doi:10.7717/peerj-cs.841)
Supplement: Supplemental Information 1 — An auto generated protege’s documentation of the proposed ontology. [file peerj-cs-08-841-s001.zip › Vro_Html/dataproperties/hasBrand___-62171973.html]

Ontology Browser


Ontologies
Classes
Object Properties
Data Properties
Annotation Properties
Individuals
Datatypes
Clouds

## Data Property: hasBrand

#### Domains (1)

- Vehicle

#### Ranges (1)

- {"Abarth", "Alfa Romeo", "Aston Martin", "Audi", "BMW", "Bentley", "Bugatti", "Cadillac", "Chevrolet", "Chrysler", "Citroën", "DS", "Dacia", "Daewoo", "Daihatsu", "Dodge", "Donkervoort", "Ferrari", "Fiat", "Fisker", "Ford", "Honda", "Hummer", "Hyundai", "Infiniti", "Iveco", "Jaguar", "Jeep", "KTM", "Kia", "Lada", "Lamborghini", "Lancia", "Land Rover", "Landwind", "Lexus", "Lotus", "MG", "Maserati", "Maybach", "Mazda", "McLaren", "Mercedes-Benz", "Mini", "Mitsubishi", "Morgan", "Nissan", "Opel", "Peugeot", "Porsche", "Renault", "Rolls-Royce", "Rover", "Saab", "Seat", "Skoda", "Smart", "SsangYong", "Subaru", "Suzuki", "Tesla", "Toyota", "Volkswagen", "Volvo"}

#### Usage (1)

- car1 hasBrand "Toyota"

OWL HTML inside
