# Supplementary material for: A forensic-driven data model for automatic vehicles events analysis
Source: PeerJ Comput Sci. 2022 Jan 5;8:e841. doi: 10.7717/peerj-cs.841 (PMC8771793; doi:10.7717/peerj-cs.841)
Supplement: Supplemental Information 1 — An auto generated protege’s documentation of the proposed ontology. [file peerj-cs-08-841-s001.zip › Vro_Html/dataproperties/HasSpeedLimit___1926697340.html]

Ontology Browser


Ontologies
Classes
Object Properties
Data Properties
Annotation Properties
Individuals
Datatypes
Clouds

## Data Property: HasSpeedLimit

#### Domains (1)

- Chekpoint

#### Ranges (1)

- {"100", "120", "40", "60", "70", "80", "90"}

#### Usage (1)

- NorthCheckpoint HasSpeedLimit "40"(xsd:string)

OWL HTML inside
