# Supplementary material for: A forensic-driven data model for automatic vehicles events analysis
Source: PeerJ Comput Sci. 2022 Jan 5;8:e841. doi: 10.7717/peerj-cs.841 (PMC8771793; doi:10.7717/peerj-cs.841)
Supplement: Supplemental Information 1 — An auto generated protege’s documentation of the proposed ontology. [file peerj-cs-08-841-s001.zip › Vro_Html/dataproperties/vehicleStatus___981546476.html]

Ontology Browser


Ontologies
Classes
Object Properties
Data Properties
Annotation Properties
Individuals
Datatypes
Clouds

## Data Property: vehicleStatus

#### Domains (1)

- Vehicle

#### Ranges (1)

- {"Nothing", "Stolen", "Wanted"}

#### Usage (1)

- car1 vehicleStatus "Stolen"

OWL HTML inside
