# Supplementary material for: A forensic-driven data model for automatic vehicles events analysis
Source: PeerJ Comput Sci. 2022 Jan 5;8:e841. doi: 10.7717/peerj-cs.841 (PMC8771793; doi:10.7717/peerj-cs.841)
Supplement: Supplemental Information 1 — An auto generated protege’s documentation of the proposed ontology. [file peerj-cs-08-841-s001.zip › Vro_Html/dataproperties/fraudType___597088108.html]

Ontology Browser


Ontologies
Classes
Object Properties
Data Properties
Annotation Properties
Individuals
Datatypes
Clouds

## Data Property: fraudType

#### Domains (1)

- Fraud

#### Ranges (1)

- {"Hit", "Licence plate fraud", "Possession of prohibited items", "Speed fraud", "Stolen", "Theft fraud", "Transportation of offenders"}

#### Usage (4)

- car1: fraudType some fraudTypeEnum
- Fraud01 fraudType "Stolen"
- isStolen Range (fraudType some fraudTypeEnum)

OWL HTML inside
